# HOSPICE USE AND END-OF-LIFE EXPERIENCE BY RACE AND GEOGRAPHY: A LATENT CLASS ANALYSIS

**DOI:** 10.1093/geroni/igad104.0245

**Published:** 2023-12-21

**Authors:** Autumn Decker, Raven Weaver

**Affiliations:** Washington State University, Pullman, Washington, United States; Washington State University, Pullman, Washington, United States

## Abstract

Long existing inequalities near the end-of-life (EOL), including the death experience, have been uncovered because of increased attention during the COVID-19 pandemic. Hospice may be an avenue to equalize EOL experiences. This study aimed to 1) understand the profiles of hospice use and EOL experience in a national representative sample of decedents and 2) uncover how race and/or geographic location predict class membership. We conducted a latent class analysis with covariates using Waves 9-11 (n=1050) from the last month of life interview in the National Health and Aging Trends Study. Based on fit statistics, response probabilities, class separation, interpretability, and fit with theory, a four-class model was ultimately selected. The four latent classes were: well-rounded hospice care; no hospice, goal concordant care; no hospice, poor overall care; and complex hospice care. We found that geographic location was not a significant predictor of latent class membership but being non-White was significantly associated (p < .001) with 8.79 times higher odds of being in the no hospice, poor overall care group and 1.85 times higher odds of being in the no hospice, complex care group compared to the reference class (well-rounded hospice care). The current study’s findings paint a complex picture of hospice use and EOL experience in a nationally representative sample. Hospice use alone does not make a “good death” experience, particularly for people of color. Rather, there are unique profiles of hospice use and experiences at EOL. Future research should explore the impact of geographic location on the death experience.

